# Neurovascular pathophysiology in cerebral ischemia, dementia and the ageing brain – current trends in basic, translational and clinical research

**DOI:** 10.1186/2040-7378-4-14

**Published:** 2012-08-10

**Authors:** Johannes Boltze, Christoph Kleinschnitz, Klaus G Reymann, Georg Reiser, Daniel-Christoph Wagner, Alexander Kranz, Dominik Michalski

**Affiliations:** 1 Fraunhofer-Institute for Cell Therapy and Immunology, Perlickstr. 1, D-04103 Leipzig, Germany; 2 Translational Centre for Regenerative Medicine, University of Leipzig, Philipp-Rosenthal-Str. 55, D-04103 Leipzig, Germany; 3 Department of Neurology, University of Würzburg, Josef-Schneider-Str. 11, D-97080 Würzburg, Germany; 4 Leibniz Institute for Neurobiology, Project Group Neuropharmacology, Brenneckestr. 6, D-39118 Magdeburg, Germany; 5 German Centre for Neurodegenerative Diseases (DZNE), site Magdeburg, Leipziger Str. 44, D-39120 Magdeburg, Germany; 6 Institute of Neurobiochemistry, Otto-von-Guericke-University Magdeburg, Leipziger Str. 44, D-39120 Magdeburg, Germany; 7 Department of Neurology, University of Leipzig, Liebigstr. 20, D-04103, Leipzig Germany

**Keywords:** Neuroprotection, Neurorepair, Cerebral ischemia, Alzheimer’s disease, Small vessel disease, Vascular dementia, Mitochondria, Astrogliosis, *in vivo* imaging, Translational research

## Abstract

The 7^th^ International Symposium on Neuroprotection and Neurorepair was held from May 2^nd^ to May 5^th^, 2012 in Potsdam, Germany. The symposium, which directly continues the successful Magdeburg meeting series, attracted over 330 colleagues from 29 countries to discuss recent findings and advances in the field. The focus of the 2012 symposium was widened from stroke and traumatic brain injury to neurodegenerative diseases, notably dementia, and more generally the ageing brain. Thereby, emphasis was given on neurovascular aspects of neurodegeneration and stroke including the blood–brain barrier, recent findings regarding the pathomechanism of Alzheimer’s disease, and brain imaging approaches. In addition, neurobiochemical aspects of neuroprotection, the role of astrogliosis, the clinical progress of cell-based approaches as well as translational hurdles and opportunities were discussed in-depth. This review summarizes some of the most stimulating discussions and reports from the meeting.

## Main text & conclusions

### Introduction and background information

Vascular disorders of the human brain are a common precondition and a relevant factor of influence for some of the most devastating neurological conditions like stroke and dementia – globally burdening patients, families, and the health care systems. Significant advances have been made regarding our understanding of central pathophysiological aspects of these diseases, as well as possible strategies to counter them. However, we yet have been unable to translate hopeful experimental findings into the clinics to augment the existing, but limited therapeutic options. In the context of ageing industrialized societies with an increasing prevalence of important risk factors like diabetes and arterial hypertension as well as the limited resources for health care worldwide, diseases affecting the cerebral vasculature are one of the most relevant global health problems. Thus, there is an increasing and still unmet need for an even better understanding of stroke and dementia in order to derive novel therapeutic approaches that clearly have the potential to be of benefit for patients.

The 7^th^ International Symposium on Neuroprotection and Neurorepair (7^th^ ISN&N) was held from May 2^nd^ to May 5^th^ at the Templin Lake in Potsdam, Germany. In continuation of the Magdeburg meeting series and the 6^th^ ISN&N [1], the meeting was organized jointly by four German institutes, the Fraunhofer Institute of Cell Therapy and Immunology (Leipzig), the Leibniz Institute for Neurobiology, the German Center for Neurodegenerative Diseases (DZNE) of the Helmholtz Society as well as Institute for Neurobiochemistry from the Medical faculty of the Otto-von-Guericke-University (all Magdeburg). A central objective of the 7^th^ ISN&N was to widen the scientific scope of the meeting series from stroke to dementia and more generally the ageing brain, primarily focusing on Alzheimer’s disease (AD) and vascular dementia. This paper reviews some of the most interesting findings being presented at the 7^th^ ISN&N and gives a compact summary of selected scientific debates that emerged during the meeting.

### A common burden: vascular aspects of neurodegeneration in stroke and dementia

Cerebral small vessel diseases are a major cause for chronic cerebral ischemia leading to cognitive decline and behavioral changes, but may also induce stroke-like symptoms if vessels in important brain regions are affected. *Holger Braun* (Magdeburg, Germany) demonstrated the sequence of events during small vessel alterations in brains of spontaneously hypertensive stroke prone rats. Accumulations of erythrocytes were shown to be the initial step in the pathological cascade, which is followed by blood–brain barrier (BBB) impairments finally leading to microbleeds that cause necrotic tissue damage in terms of microinfarcts [2]. Interestingly, the progress of small vessel alterations simultaneously develops in the kidney, demonstrating that the cerebral microangiopathology is part of a systemic vessel disease in those animals. Clinical research and experimental work suggests that cerebral blood flow is impaired in AD, what may contribute to the detrimental cognitive effects. *Nozomi Nishimura* (Ithaca, USA) hypothesized that inflammation could play a role in such reductions in blood flow in AD by leukocyte plugging of capillaries. *In vivo* two-photon excited fluorescence microscopy was used to examine cortical blood flow in mouse models of AD. To determine whether or not individual brain microvessels are stalled, the movement of non-fluorescent blood cells within the dye-labeled blood plasma was monitored [3]. Thereby, several capillaries were found to plug longer by leucocytes in AD mice compared to wild type mice. Additional evidence for remodeling of the cerebral microvasculature in AD comes from magnetic resonance angiography. *Didier Leys* (Lille, France) provided a review of stroke and dementia as consequences of an altered vasculature, which was followed by a lively round table discussion.

### An interesting ‘frontier’: targeting the blood–brain barrier integrity

In recent years, the BBB received increasing attraction in stroke and neurodegeneration, since the concept of a primary separating role (for instance leading to edema and hemorrhage when the BBB integrity is lost) was added by a functional perspective including inflammatory aspects [4,5]. Thereby, mediators like cytokines contribute to leukocyte migration, maintaining the inflammatory response with predominantly damaging effects [6]. Despite the uncertainty of the exact cellular composition forming the BBB along the vascular tree [7], the neurovascular unit (NVU) represents an accepted concept that embeds the BBB into the network of microvessels, glial cells and neurons [4,8]. *Gregory J. del Zoppo* (Seattle, USA) presented an overview on the barrier structure within the NVU: Since previous research predominantly addressed inter-endothelial cell tight junction complexes, growing evidence indicated that also the adhesion between endothelial cells and the extracellular matrix (arranged amongst others by β1-integrin) contribute to BBB permeability [9]. This perspective is supported by the recently demonstrated increase in IgG extravasation after intrastriatal injection of an antibody inhibiting the function of β1-integrin in mice [10]. *Berislav V. Zlokovic* (Los Angeles, USA) stressed the NVU with emphasis on neurodegenerative processes, in particular AD. Thereby, a reduced clearance of β-amyloid peptides is assumed to emerge from BBB alterations, contributing to neuronal dysfunction and finally dementia [5]. In this context, pericytes were found to act as key players in BBB regulation with the potency to promote both BBB breakdown and integrity [11], thus representing a target for future research. While extending this perspective by glial cells, recent research yielded that different isoforms of apolipoprotein E, originating from astrocytes, have opposite effects on the release of matrix metalloproteinase-9 (MMP-9), an enzyme known to mediate BBB breakdown [12]. *Dominik Michalski* (Leipzig, Germany) focused on side effects of recombinant tissue plasminogen activator (rtPA), currently representing the best available drug-related treatment in acute stroke. Despite the hypothesis that detrimental actions could be attenuated by co-administered hyperbaric oxygen, recent findings failed to support this theoretically forced approach [13]. However, further research is required to develop neuroprotective strategies which can be applied simultaneously with rtPA. *Mariel G. Kozberg* (New York, USA) introduced new approaches to characterize the NVU by *in vivo* imaging in rodents. Thereby, optical-based techniques were applied to provide information on blood flow, vessel dilations and rate of oxygenation in different types of vessels and the parenchyma. Two-photon microscopy was found to image neurovascular coupling by using intravenously applied agents for concomitant labeling of cells and vessels. *Guido Stoll* (Würzburg, Germany) compared different contrast agents for detecting BBB dysfunction in magnetic resonance imaging (MRI), whereas gadofluorine M was found to best characterize the size of injury after cortical ischemia in mice [14]. Further, the link between BBB disruption and inflammation was established with MRI enhanced by gadolinium-diethylenetriamine penta-acetic acid (DTPA) and superparamagnetic iron oxide particles (SPIOs). Thereby, infiltrating macrophages as visualized by SPIOs were found to occur within the area of impaired BBB integrity after cortical ischemia [15], which was not a requested condition in experimental autoimmune encephalomyelitis [16]. These data indicate that the infiltration of inflammatory cells is not compulsorily associated with BBB opening as indicated by leakage of gadolinium-DTPA or gadofluorine M.

### Small molecules with large impact I: new insides into the role of Aβ in Alzheimer’s dementia

While focusing on AD as a major topic of the 7^th^ ISN&N, *Hans-Ulrich Demuth* (Halle, Germany) emphasized that toxicity in AD is induced by pyroglutamate (PGLU)-Aβ, and furthermore, depends on tau protein. Demuth demonstrated that the cytotoxicity is propagated by a prion-like templating mechanism of Aβ misfolding initiated by PGLU-Aβ and that these PGLU-Aβ-containing low-molecular weight oligomers remain stable for days - in contrast to oligomers not formed by the PGLU-Aβ initiated mechanism [17]. *Jens Pahnke* (Rostock and Magdeburg, Germany) reported that the BBB transporter ABCC1 has an important role in cerebral Aβ clearance and accumulation. Moreover, a pharmacological activation of ABC transporters can slow down the neurodegenerative cascade [18].

### Small molecules with large impact II: underlying mechanisms of neuroprotective approaches in stroke, ageing and neurodegenerative diseases

Within the numerous efforts to develop novel or even to improve existing neurochemical approaches, topical sessions of the 7^th^ ISN&N focused on promising molecular targets and on mitochondrial processes. The role of a transcription factor family that is denoted as peroxisome proliferator-activated receptor (PPAR) was presented by several speakers. First, *Douglas Feinstein* (Chicago, USA) reported on the potential use of PPARγ-sparing thiazolidinediones for the treatment of neurodegenerative diseases. These substances, also known as glitazones, are a class of drugs already used in the treatment of diabetes mellitus type 2. Second, *Michael Heneka* (Bonn, Germany) described PPARγ as a mediator of protection in AD, and third, *Anne Murphy* (La Jolla, USA) dealt with the value of a preclinical systematic review to analyze mitochondria as targets of thiazolidinediones. In addition, *Georg Reiser* (Magdeburg, Germany) concentrated on the role of mitochondria to describe a major principle for neuroprotection. His report covered the permeability transition in mitochondria [19] and thereby described a second neuroprotective route: the activated protein C / endothelial protein C receptor signaling system, which was shown as a promising target in several stroke models. Finally, *Raghu Vemuganti* (Madison, USA) reported that ubiquitin-like protein modifications are neuroprotective after stroke.

### Seeing is believing: novel approaches in imaging the affected brain

*In vivo* imaging of structures and processes in living animals as disease models is commonly used to directly prove a stated experimental hypothesis. In the context of continuous technical advances in imaging techniques and analyzing tools, this research field qualifies to solve two major tasks: imaging of direct functional adaptation and changes in plasticity after brain injury, and further, to characterize the morphological structures on microarchitectural levels. During the meeting, the latest studies which focused on the development of MRI, positron emission tomography (PET) and live cell imaging for this purpose were discussed. *Alan Koretsky’s* group (Bethesda, USA) recently used high field MRI to distinguish the arterial and venous hemodynamic response in individual intracortical vessels after whisker pad stimulation [20]. By using high spatial (150 μm) and temporal (200 ms) resolution they described the spatio-temporal pattern of the blood oxygenation level dependent (BOLD) fMRI signal and clarified the influence of the micro- and macrovascular architecture. *Rick Dijkhuizen* (Utrecht, The Netherlands) brought new insides in the reorganization and functional recovery after stroke by comparing graph-based analyses of fMRI signals in gray matter, quantification of fractional anisotropy in white matter by diffusion tensor imaging (DTI) and functional recovery in relation to stroke severity [21]. The group was able to show significant correlations between increased functional connectivity (especially in the area of the sensorimotor cortex) augmented fiber tracts – mainly in the ipsilesional corticospinal tract –and better outcome in the sensorimotor performance score. Using contrast-enhanced microangiography, *Jan Klohs* (Zurich, Switzerland) showed an age-dependent reduction in the number of functional intra-cortical microvessels in aged (24 months) arcAβ mice. This reduced density of transcortical vessels (with radii less than 20–80 μm) was attributed to an impaired perfusion and vascular occlusion caused by deposition of Aβ and fibrin [22]. The Florbetaben study, a multicenter phase II study which could identify amyloid-β-PET as a visual adjunct for the routine diagnostic of dementia, was presented by *Henryk Barthel* (Leipzig, Germany) representing a translational approach which impressively linked preclinical and clinical research [23]. As an example for clinical stroke research, *Marc Fisher* (Worcester, USA) summarized clinical trials which used imaging criteria as a decision instrument for the application of recanalization therapies. It was shown that especially MRI is useful to identify subgroups which clearly benefit from a consecutive thrombolytic therapy, also at comparatively late stages.

### Friend or foe: the immune system in post-stroke pathophysiology

The interesting role of the immune system in the pathophysiology of cerebral ischemia was highlighted in the session ‘Balancing Immune Responses in Neurodegeneration’. *Roland Veltkamp* (Heidelberg, Germany) emphasized the ‘Janus-faced’ nature of T cells in stroke development. While several groups could meanwhile confirm that CD4^+^ and CD8^+^ T cells act detrimental during the acute phase of stroke, the contribution of other T cell subsets, in particular regulatory T cells (Treg), remains controversial. In line with the description of a beneficial role of Treg for stroke recovery and repair, recently published by his group, Veltkamp now outlined a promising approach to pharmacologically enhance Treg immune functions in stroked mice. In contrast, the group of *Christoph Kleinschnitz* (Würzburg, Germany) failed to confirm a neuroprotective effect of Treg during the course of cerebral ischemia. By using a mouse model of selective FoxP3^+^ Treg ablation they could even identify Treg as key mediators of ischemic neurodegeneration – at least during the early phase after transient middle cerebral artery occlusion [24]. As potential reasons for these opposed findings different stroke models and immunological tools were lively discussed by the speakers and audience. Thereby, *Ulrich Dirnagl* (Berlin, Germany) took this controversy as a good example to stress the need for randomized, multicenter preclinical stroke trials. Further emphasis was placed on the role of polymorphonuclear leukocytes and microglia in stroke. *Emmanuel Pinteaux* (Manchester, UK) pointed out that central and peripheral inflammations represent key drivers of tissue damage in response to acute brain injury such as stroke, and are associated with poorer outcome in stroke patients. He demonstrated that interleukin (IL)-1 is primarily expressed by activated microglia, and acts on astrocytes to release MMP-9, known to contribute on neuronal cell death. IL-1 was also found to activate the brain endothelium leading to neutrophil infiltration into the brain and neuronal death, a mechanism that is associated with neutrophil MMP-9 activity and the degradation of the extracellular matrix [25]. Investigating the penumbra by intracranial two-photon microscopy, *Jens Neumann* (Magdeburg, Germany) revealed an elevated slow rolling of neutrophil granulocytes on the endothelium followed by an infiltration of these cells into the brain. Contradicting data were shown by *Britta Engelhardt* (Bern, Switzerland), who provided evidence that neutrophil granulocytes do not enter the parenchyma but stay in close proximity to the vessels.

### More than just a scar: the Janus face of astrogliosis

Reactive astrogliosis has long been identified as a simple scarring process in the central nervous system (CNS) that ultimately impairs neural regeneration. However, recent evidence challenges this paradigm, notably by combining genetic ablation of intermediary filaments with different models of CNS diseases. Hence, the scientific community is now reconsidering the unidirectional view on reactive astrocytes and is developing a more differentiated approach. *Michael V. Sofroniew* (Los Angeles, USA) presented a comprehensive overview on the role of astrocytes in the normal and damaged CNS and particularly emphasized the morphological and functional heterogeneity of reactive astrogliosis. Beyond reactive astrogliosis and its implications, astrocytes might also contribute to CNS disorders either by a loss of normal, or a gain of detrimental functions. *Sofroniew* concluded that the understanding of these processes might uncover novel therapeutic targets. *Magdalena Götz* (Munich, Germany) highlighted the potential beneficial action of reactive astrocytes to develop neural stem cell properties beyond the classical CNS niches. These characteristics can be observed after different types of CNS injury and are principally regulated by Sonic hedgehog signaling. *In vivo* imaging of reactive astrogliosis using two-photon microscopy supported the observation of astrocytic heterogeneity: while many astrocytes remained unchanged after focal CNS damage, a subpopulation of astrocytes showed proliferation within the vascular niche. *Milos Pekny* (Gothenburg, Sweden) first introduced a novel three-dimensional culture system for astrocytes that largely preserved the complex cellular morphology and reduced the baseline activation which is a common drawback of conventional astrocyte cultures. *Pekny* further provided evidence for a differentiated role of reactive astrogliosis with beneficial effects in the context of AD and Batten disease, but a detrimental action during amyotrophic lateral sclerosis. Finally, he proposed that reactive astrogliosis and its consequences are disease-specific. Additionally, *Karsten Ruscher* (Lund, Sweden) linked a common treatment of Parkinson’s disease with stroke while reporting on the beneficial effects of levodopa on functional recovery after stroke. As a conclusion, he hypothesized that peri-infarct astrocytes equipped with dopamine receptors may contribute to these effects.

### Entering the clinics: recent progress in stem cell-based research

For almost two decades, cell- and stem cell-based approaches are considered among the most promising options to treat degenerative diseases of the brain for which regenerative treatments have not been available so far. Even though cell-based therapies have not been proven beneficial in human patients, first data are available from clinical phase I and II trials. In parallel, more concepts emerged to late preclinical phases. Induced pluripotent stem cells (iPSC) were reported to have capacities comparable to those of embryonic stem cells, but can be generated from autologous, patient-specific somatic cells [26]. *Brigitte Onteniente* (Paris, France) showed that intracerebrally administered human iPSC-derived neural progenitor cells differentiate into astrocytes, endothelial cells and GABAergic neurons after transient cerebral ischemia in the rat, ameliorating motor deficits and secondary neurodegeneration. *Robert Mays*, cofounder and Senior Director of Athersys Inc. (Cleveland, USA) introduced the company’s multipotent adult progenitor cell (MAPC) program, which currently includes clinical phase II testing. MAPC were shown to improve functional recovery and to act neuroprotectively by promoting immune system homeostasis following experimental stroke. An open-label, single arm clinical phase I study using intravenous administration of autologous bone marrow mononuclear cells (BM MNC), which also have been thoroughly investigated in preclinical experiments, was conducted by *Sean Savitz* (Houston, USA) and colleagues [27]. Obtaining 1x10^7^ BM MNC per kilogram bodyweight by iliac crest puncture as well as subsequent density gradient centrifugation and reinfusion of the cells within 72 hours following stroke onset were shown both feasible and safe in 10 patients. A clinical phase IIb study is actually being prepared, also assessing efficacy endpoints. Intraparenchymal stem cell administration is considered beneficial even in subacute and chronic stages after cerebral ischemia [28,29]. The Department of Neurosurgery at Stanford University participates in numerous early stage clinical trials for intraparenchymal administration of various stem cell types. *Gary Steinberg* (Palo Alto, USA) reported encouraging safety results from these trials. In summary, cell-based approaches have tremendously advanced during the last years now being under clinical evaluation. However, it is still unsure whether these approaches can overcome the translational roadblock and show efficacy in human stroke.

### Roadblocks, bypasses, and alternative directions: how to move on in translational research?

Virtually all approaches to transfer experimental therapies for stroke and dementia into the clinics have failed so far, mainly due to a lack of efficacy and/or safety. While the reasons for this dilemma (being referred to as the ‘translational roadblock’) are still a matter of debate, it appears likely that this roadblock will be hard to overcome [4]. *Wolf-Rüdiger Schäbitz* (Bielefeld, Germany) reported on the most recent drug candidate that got stuck on its way into the clinic. Granulocyte colony-stimulating factor (G-CSF), although being carefully evaluated in experimental studies [30] and proven safe in a phase IIa trial [31], recently failed to be effective in the phase IIb AXIS 2 study. Schäbitz also drew relevant conclusion of what may be improved to prevent such failures in future studies. Particularly, the patient population heterogeneity needs to be taken into account by testing the influence of age, comorbidities and polypharmacology in preclinical studies, also using large animal models to more closely mimic the human condition. On the other hand, there is no good reason to believe that the translational failure is an inevitable event. *Ulrich Dirnagl* gave some very interesting examples for relevant results that were ‘found in translation’, thereby proving evidence that preclinical stroke research can be predictive. For example, the penumbra concept was first reported in primate studies and was reconstructed in rodent models while the time window for intravenous thrombolysis [32] as well as beneficial effects of statins on stroke outcome closely resemble in preclinical and clinical studies. Dirnagl also suggested novel concepts for a more predictable experimental research such as multi-center, international ‘phase III’ preclinical trials. We still need, however, to find out what particularly prevents so many research activities to be predictive for the human situation.

## Conclusions

The 7^th^ ISN&N attracted more than 330 scientific colleagues from 29 countries on 6 continents to discuss recent findings and novel concepts in several types of brain injury. Personal comments of numerous attendees, a growing interest in the meeting’s topics indicated by steadily increasing numbers of participants as well as very stimulating discussions emerging throughout the meeting underlined the unbroken demand for a forum tailored to discuss our research in the addressed fields. Consequently, the 8^th^ International Symposium on Neuroprotection and Neurorepair will take place from April 9^th^ to April 12^th^, also going back to the roots of the meeting series in Magdeburg, Germany – were the series originally was started in 1998. The 8^th^ ISN&N will strengthen the focus on pathophysiological aspects of the altered vasculature in the ischemia-affected and ageing brain.

## Abbreviations

AD, Alzheimer’s disease; BBB, Blood–brain barrier; BM MNC, Bone marrow mononuclear cells; BOLD, Blood oxygenation level dependent; CD, Cluster of differentiation; CNS, Central nervous system; DTI, Diffusion tensor imaging; DTPA, Diethylenetriamine penta-acetic acid; DZNE, Deutsches Zentrum für Neurodegenerative Erkrankungen; G-CSF, Granulocyte colony-stimulating factor; IL, Interleukin; iPSC, Induced pluripotent stem cells; IgG, Immunoglobulin G; ISN&N, International Symposium on Neuroprptection & Neurorepair; MAPC, Multipotent adult progenitor cell; MMP-9, Matrix metalloproteinase-9; MRI/fMRI, (functional) magnetic resonance imaging; NVU, Neurovascular unit; PET, Positron emission tomography; PGLU, Pyroglutamate; PPAR, Peroxisome proliferator-activated receptor; rtPA, Recombinant tissue plasminogen activator; SPIO, Superparamagnetic iron oxide particle; Treg, Regulatory T cell; UK, United Kingdom; USA, United States of America.

## Competing interests

JB, KR, GR, DCW and AK were organizers of the 7^th^ ISN&N; GR, DM and CK were invited speakers to the conference. None of the authors has to disclaim any other, particularly commercial or financial competing interests.

## Author’s contributions

All authors contributed actively to the conception, writing and revision of the manuscript, and approved its final version. JB coordinated the writing process and was the conference president of the 7^th^ ISN&N.
